# Making implementation science more efficient: capitalizing on opportunities beyond the field

**DOI:** 10.1186/s13012-023-01298-9

**Published:** 2023-09-11

**Authors:** Michel Wensing, Paul Wilson

**Affiliations:** 1 Heidelberg University Hospital, Heidelberg, Germany; 2 Centre for Primary Care and Health Services Research, University of Manchester, Manchester, UK; 3 NIHR Applied Research Collaboration Greater Manchester, Manchester, UK

**Keywords:** Implementation science, Research methodology, Research policy, Health services research

## Abstract

Implementation researchers often find themselves as research partners in practice improvement projects, clinical trials or other applied health studies. The implementation science component in these projects can be described as supportive, descriptive or explanatory. This commentary reflects on the potential contributions of such projects to implementation science. They may provide evidence on implementation strategies, so it is essential to identify and evaluate these separately from the clinical and preventive interventions of interest. The use of theory on implementation processes and associated factors can contribute to knowledge accumulation, particularly if the focus is on what actually gets implemented when, why and how. The development and validation of relevant measures is a third potential contribution to implementation science. Although not all issues in implementation science can be addressed in this way, capitalization on the opportunities beyond the field can contribute to implementation science.

Contributions to the literature

- Implementation scientists frequently support projects by helping to choose and design implementation strategies, contribute to the evaluation of implementation processes and provide insights into outcomes.
- Projects outside implementation science can contribute to the field, if implementation researchers embed research concerning implementation strategies, processes and associated factors.
- Nevertheless, some research questions, such as those concerning the effectiveness of implementation strategies, require dedicated implementation research.

## Introduction

Research on the implementation of innovations and recommended practices in healthcare has attracted increasing interest across the world. The items of interest are, for instance, clinical practice guidelines, health technologies and service delivery models. Implementation research has many flavours, varying from formative research in a healthcare organization to multi-centre randomized trials. In practice, implementation researchers often find themselves working as research partners in practice improvement projects, clinical trials or other applied health studies. In this role, they can help to provide theoretically informed frameworks for evaluation, choose and design implementation strategies, document their delivery in practice, explore implementation processes and associated factors and provide contextual insights into differences between anticipated and observed outcomes. In short, the involvement of implementation scientists can strengthen both implementation practices and evaluation approaches in projects. The main objectives of these projects concern achieving actual improvement in practice or evaluation of intervention effectiveness. These projects may be fruitful for implementation science by pointing to issues that are of interest to the field; in this sense, the partnership can be bidirectional. Nevertheless, from an implementation science perspective, the implementation science component in these projects is often limited and best characterized as supportive, descriptive or explanatory.

In many calls for research, the health content and political priority of topics seems predominant in the decision to fund a project application. Some calls of research are actually labelled as implementation research, although the emphasis remains on the study of effectiveness of clinical or preventive interventions. Examples can be found among research programs of the European Community as well as national funders. Nevertheless, it can be observed that many calls for research nowadays include requirements to integrate implementation activities and/or implementation research in the research enterprise. Whilst funding opportunities for studies that have a primary focus on implementation science do exist, these are very much in the minority. Most implementation researchers are therefore engaged in work beyond the primary focus of the field. How can they best make use of these opportunities and contribute knowledge to implementation science? This commentary reflects on this topic and offers a number of suggestions. Before this, it will elaborate on what type of studies actually contribute most knowledge to implementation science in health.

### Knowledge accumulation in implementation science

The accumulation of scientific knowledge on the implementation of innovations in health settings is slow [1]. This is caused by range of factors, such as the absence of implementation science concepts in studies, the use of weak research methods and the limited contextualization of studies in the implementation science literature. Three types of studies are particularly relevant for knowledge accumulation in the field of implementation science:

- Meaningful, rigorous and generalizable evaluation of the effectiveness of implementation strategies;
- Analytical, theory-guided studies of implementation processes and determinants of implementation outcomes (e.g. contextual factors or innovation characteristics);
- Studies that develop and validate measures that are relevant for implementation science.

This paper takes an inclusive approach to implementation research. It covers uptake, adoption, translation, dissemination, scale-up, knowledge transfer, knowledge utilization, and sustainment, as well as stopping practices that are no longer desired (de-implementation). Whilst these concepts differ in subtle ways, the commonalities are substantial.

Implementation strategies are educational, behavioural, organizational, financial or regulatory interventions to enhance the adoption of innovations of interest in a targeted population, typically healthcare providers or other decision-makers. Evaluations of implementation strategies (type A as listed above) are meaningful, if these fill gaps in scientific knowledge. Planned replication of research can be useful, but unnecessary replication does not enhance knowledge. Regular synthesis of the available research evidence, using systematic methods, should therefore guide the planning of research. Studies are rigorous if they provide a high certainty that the study results are valid, which is influenced by the study design, conduct and interpretation of studies. Generalizability is influenced by a range of characteristics, including representative, sufficiently large samples of healthcare providers; patient populations and healthcare delivery contexts that reflect routine practice rather than optimized conditions; and a degree of flexibility in the delivery of the implementation strategies that reflects real world rather than optimized conditions.

Implementation research does not only concern strategies for implementation but also the context in which implementation is supposed to happen, the innovation characteristics that influence implementation and the implementation mechanisms of action. These issues can be broadly described as implementation processes and associated factors. The use of theory in empirical studies on these topics (type B) is another way of systematically contributing to knowledge accumulation as well as building on available scientific knowledge. Theory-driven research can take different approaches. It can provide rigorous, reproducible frameworks to guide evaluation and knowledge accumulation. It can map identified implementation-related factors and processes onto implementation science frameworks. Studies can also include measures of specific concepts, typically questionnaires that are completed by healthcare providers, and then examine their hypothesized impacts empirically. Research may also use qualitative data to theorize on implementation-related processes and factors by linking these to theories from various scientific disciplines [2]. The actual contribution to implementation science is dependent on adequate selection and operationalization of theories, considering the body of available research evidence.

Validation of measures (type C) is essential as implementation science is short of validated measures, like health services research generally. Whilst many questionnaires, interview guides and data-abstraction tools are available, most are not well validated and used only once [3]. The development of quantitative data-analysis methods (e.g. tools that artificial intelligence) has by far exceeded the development of validated measures in implementation science.

### Implementation strategies

Implementation researchers who work in projects outside implementation science are often expected to contribute to intervention development and conduct process evaluation of these interventions in practice. A first step is to specify implementation strategies separately as they are often included in packages of complex interventions. Implementation strategies are not always labelled as such but in various other ways, such as continuing education and quality improvement. It is thus important to recognize and describe these strategies in order to be able to contribute to implementation science. As implementation strategies are described inconsistently, it is important to map the identified strategies onto standardized terminology whenever possible. Whilst the classification of implementation strategies is under development, the best choice at this moment is probably the ERIC list of implementation strategies (despite its logical incoherence and incompleteness in some respects) [4]. Further specification of the strategies is recommended for research purposes, considering aims, ingredients, mechanisms, and delivery formats [5]. Unfortunately, there is little guidance on how to specify these aspects, with the exception of psychological interventions for which behavioural change techniques have been specified [6]. Finally, to aid further reproducibility, logistical and practical aspects should also be documented in sufficient detail, using a reporting guideline such as TIDIER [7].

As most clinical and public health trials focus on health outcomes in patients and populations, outcomes that reflect the effects of implementation strategies may need to be added (typically as secondary outcomes). These are usually aspects of health professionals’ behaviours or healthcare delivery processes, such as medication prescribing or test ordering. They may be measured in routinely recorded data or with the use of structured questionnaires. In the causal chain of effects, professional behaviours and healthcare delivery processes precede and influence health outcomes of individuals and populations. Perceptions of new practices and implementation strategies are also informative but cannot replace actual implementation outcomes.

Implementation research usually focuses on healthcare providers. However, professional behaviours are best measured in relation to individual patients (e.g. number of eligible patients with recommended procedures done). In practice, clinical studies are often optimized in terms of samples of patients, which may imply that few and non-representative healthcare providers are available for implementation research. In addition, implementation strategies are applied in clinical trials to optimize adoption of interventions, which may result in the use of strategies that have already been extensively studied (e.g. continuing medical education [8] or feedback to providers [9]). The added value of the evaluation of implementation effectiveness may thus be limited. Whilst it is rarely possible to include a randomized comparison between implementation strategies in a non-implementation science project, it may be possible to conduct non-randomized comparisons among subgroups in the trial or between trial participants and non-trial participants—in effect, a study within a trial (SWAT) [10].

An example of such a SWAT can be found in the ARENA project, which was a quality improvement project to reduce unnecessary use of antibiotics in respiratory tract infections [11]. Whilst all primary care practices in the project received additional reimbursement, training and feedback on prescribing patterns, one group also received interventions for practice assistants and another group received also received a computerized decision support tool. Randomization occurred at the level of practice networks, in which these practices were embedded. In addition, an observational comparison between study participants with primary care practices outside the study was planned. The study found no difference between randomized groups but a reduction of antibiotics prescribing rates among all study participants compared to practices outside the project. The ARENA project suggested that any package of implementation strategies improved practice as compared to usual care, whilst the exact composition of the package seemed less relevant.

### Implementation processes and associated factors

A theory-informed analysis of the process of implementation and its determinants should be informed by the implementation science literature. Knowledge beyond a single implementation science framework, and in fact beyond frameworks generally, is often required for this. Such an analysis should go beyond descriptive lists of determinants and focus instead on causal mechanisms through which implementation or contextual variables influence observed outcomes [12]. In most cases, relevant theories are available both within and beyond the field and should be used, rather than inventing new ones. Some concepts have been extensively studied (e.g. organizational readiness for change) and may thus not offer a route to knowledge accumulation. It is generally wise to focus on a few concepts and issues in a specific project, rather than (only) do a broad exploration.

Both quantitative and qualitative research methods may be used. The ARENA project, which was mentioned above, included a mixed-methods process evaluation, based on semi-structured interviews and repeated written surveys among participating primary care physicians [13]. This study showed, for instance, that some implementation strategies (e.g. financial incentives) were only used by some practices. Physicians felt that participation in the practice networks was a major facilitator of implementation, because these motivated for adherence to clinical practice guidelines. In line with this finding of the structured surveys, the semi-structured interviews confirmed the central role of the Theoretical Domains Framework-domains ‘environmental context and resources’, ‘social/professional role and identity’ and ‘beliefs about consequences’.

### Validation of measures

Of interest to implementation science are measures of implementation outcomes and of associated factors or determinants. Implementation outcomes are indicators of implementation success of failure; these may be anticipated or actual outcomes [14]. Actual outcomes include observed changes in professional behaviours and healthcare delivery processes, which can directly affect health outcomes of patients and populations. These include, for instance, reach and penetration in a targeted population, uptake and adoption of practices (e.g. guideline adherence), scale-up and sustained adoption. Cost and equity may also be covered by measures of implementation outcomes. Determinants of implementation outcomes include a wide variety of factors, varying from individual provider characteristics and perceptions of implementation to organizational and financial factors. For instance, many questionnaires on potential determinants of implementation were identified in a systematic review [15].

Validation of measures for implementation science (or beyond) is rarely separately funded, so a pragmatic approach is often required. Validation studies may be integrated in other research projects or conducted without specific funding. It is probably wise to find a middle way between methodological rigour and pragmatism. For instance, small changes in a validated questionnaire may be acceptable with limited testing rather than a new validation study. Implementation science as a field would also benefit from new types of measures beyond questionnaires, interviews and extraction from medical records. Examples in other fields (e.g. spectrometry, genetic tests, or functional magnetic resonance imaging) have shown that such measures can change scientific fields fundamentally. The need for measures is enhanced by modern data analysis methods, which are increasingly based on artificial intelligence, for which projects outside implementation science might offer opportunities.

### Limitations of non-implementation research projects

It can be challenging to include implementation research in projects. The person asked to help bring implementation science perspective to a project may find it difficult to propose an embedded project, especially if funding is tight and/or if they are junior. In addition, some research questions cannot be answered in projects outside the field of implementation science. For rigorous evaluation of the effectiveness of implementation strategies, cluster randomized trials and related study designs are required in which implementation strategies are head-to-head compared with alternative strategies or no strategy for implementation. This is rarely possible within clinical and public health trials of interventions as implementation research requires that all participants are encouraged to apply the clinical or preventive interventions of interest whilst they receive different implementation strategies. In terms of study population, sample size calculation, intervention delivery and other aspects, implementation trials need to be optimized with respect to the evaluation of implementation strategies rather than the clinical or prevention strategies. The simultaneous assessment of clinical and implementation effectiveness in so-called hybrid effectiveness-implementation trials is therefore challenging [16]. For instance, the sample of healthcare providers is small in many clinical studies (e.g. fewer than 10–15 clinicians), and the inclusion of secondary outcome measures (which may be essential for implementation science questions) is often restricted to reduce burden for study participants.

In theory-guided studies of implementation processes and associated factors, it is often essential to apply purposive or stratified sampling of participants to enhance the informativeness and generalizability of findings. For instance, a study may require both organizations with high readiness for change and organizations with low readiness. In many studies, however, the recruitment of participants is determined by considerations such as the likelihood that an innovation is accepted and applied. In addition, only intervention arms may be available for implementation research in order to avoid improvement of practice due to attention of researchers (the Hawthorne-effect). These issues reduce the informativeness and generalizability of findings of implementation research within the context of clinical trials and other projects.

## Discussion

Table 1 summarizes the potential contributions of non-implementation science projects to the field. The role of implementation researchers in some research projects is similar to that of statisticians, ethicists and health economists in those projects. From the perspective of the principal investigators, they provide support services. They all need to be creative within limitations of the projects to enhance their own scientific field or find other ways to enhance their field outside these projects. A major difference may be that these disciplines use simulated data or theoretical reflection to enhance their fields, whilst implementation science needs real-world data. Theoretical reflection and simulation modelling can only partly complement this.

**Table 1:** Examples of how non-implementation science projects can provide opportunities for contributions to the field

	**Clinical or public health trials**	**Practice improvement projects (without research or evaluation)**	**Other applied health studies**
Implementation strategies	Description of fidelity and adaptation of strategies in practice; assessment of potential impacts; opportunities to conduct SWATs	Provide ideas on or identify potential knowledge gaps for implementation strategies on the basis of real-world experiences	Description and analysis of observed strategies
Implementation processes and associated factors	Analytical, theory-guided exploration in process evaluation and related research; opportunity to generate theoretically informative research and mechanism-based explanations that can advance conceptual knowledge of the field	Provide ideas on processes and factors on the basis of real-world experiences	Description and exploration of processes and factors; opportunity to generate theoretically informative research and mechanism-based explanations that can advance conceptual knowledge of the field
Validation of measures	Embedded validation studies	-	Embedded validation research

This commentary offered a number of reflections and suggestions to implementation researchers who are involved in projects, which are not primarily focused on implementation science. It did not discuss the research funding system at large, which overall has few incentives for implementation science [1]. Also, we did not focus on what implementation researchers might contribute to projects or the practical experiences that these projects offer to them, which may result in ideas for implementation research.

If we are to advance knowledge accumulation in the field, there may be a need to do this in a coordinated way. The Trial Forge collaboration is an exemplar model that has sought to reduce research waste by taking a systematic approach to making trials more efficient by embedding opportunities to develop, refine and test trial methodologies within a research project [17]. Whilst efforts have been made to establish implementation laboratories, some of these have been narrowly focused on dedicated implementation research studies and around a single implementation strategy [18]. An opportunity to develop a similar initiative for implementation science and one that capitalizes on activity beyond the field may therefore exist. Efforts could focus on collating what is already known, highlighting gaps in current knowledge and identifying key priority areas that may benefit from further methodological, theoretical or empirical enquiry.

In an ideal world, analytical, descriptive, explorative and supportive implementation research influence each other in a fruitful way. Supportive implementation research provides societal and practice legitimacy to the field and enhances the embeddedness of implementation science in real-world healthcare. Nevertheless, analytical studies dedicated primarily to implementation science remain essential for knowledge accumulation and innovation of the field. Supportive implementation research builds on such research and some research questions can only be answered in this way.

## Data Availability

Not applicable.
